# Multiple-stage decisions in a marine central-place forager

**DOI:** 10.1098/rsos.160043

**Published:** 2016-05-11

**Authors:** Ari S. Friedlaender, David W. Johnston, Reny B. Tyson, Amanda Kaltenberg, Jeremy A. Goldbogen, Alison K. Stimpert, Corrie Curtice, Elliott L. Hazen, Patrick N. Halpin, Andrew J. Read, Douglas P. Nowacek

**Affiliations:** 1 Department of Fisheries and Wildlife, Marine Mammal Institute, Hatfield Marine Science Center, Oregon State University, Newport, OR, USA; 2 Division of Marine Science and Conservation, Duke University Marine Laboratory, Beaufort, NC, USA; 3 Savannah State University, Savannah, GA, USA; 4 Hopkins Marine Station, Department of Biology, Stanford University, Pacific Grove, CA, USA; 5 Moss Landing Marine Laboratory, Moss Landing, CA, USA; 6 NOAA Environmental Research Division, Monterey, CA, USA

**Keywords:** diving, foraging decisions, predator–prey interactions

## Abstract

Air-breathing marine animals face a complex set of physical challenges associated with diving that affect the decisions of how to optimize feeding. Baleen whales (Mysticeti) have evolved bulk-filter feeding mechanisms to efficiently feed on dense prey patches. Baleen whales are central place foragers where oxygen at the surface represents the central place and depth acts as the distance to prey. Although hypothesized that baleen whales will target the densest prey patches anywhere in the water column, how depth and density interact to influence foraging behaviour is poorly understood. We used multi-sensor archival tags and active acoustics to quantify Antarctic humpback whale foraging behaviour relative to prey. Our analyses reveal multi-stage foraging decisions driven by both krill depth and density. During daylight hours when whales did not feed, krill were found in deep high-density patches. As krill migrated vertically into larger and less dense patches near the surface, whales began to forage. During foraging bouts, we found that feeding rates (number of feeding lunges per hour) were greatest when prey was shallowest, and feeding rates decreased with increasing dive depth. This strategy is consistent with previous models of how air-breathing diving animals optimize foraging efficiency. Thus, humpback whales forage mainly when prey is more broadly distributed and shallower, presumably to minimize diving and searching costs and to increase feeding rates overall and thus foraging efficiency. Using direct measurements of feeding behaviour from animal-borne tags and prey availability from echosounders, our study demonstrates a multi-stage foraging process in a central place forager that we suggest acts to optimize overall efficiency by maximizing net energy gain over time. These data reveal a previously unrecognized level of complexity in predator–prey interactions and underscores the need to simultaneously measure prey distribution in marine central place forager studies.

## 1. Introduction

Animals have evolved multiple strategies to optimize searching for prey and maximize net energy gain with respect to costs associated with acquiring energy [1]. Charnov's [2] initial treatise developed models for how predators forage when prey are distributed in patches, and how predators must make decisions regarding when to leave a patch and travel to a new one. The marginal value theorem posits that predators should exploit individual patches until the rate of gain diminishes to the average for the environment. However, as the distance between patches increases, so does travel time, and therefore the optimal amount of time to remain in a patch predictably increases. There are voluminous examples in the literature about optimal foraging in terrestrial predators and the factors that influence this strategy. Two pertinent classic examples for our study are (i) the concept of central place foraging [3–5], where foraging rates and energy gain are affected by the need to come and go to a centrally located place (e.g. nest) and (ii) foraging and conflicting demands, where the foraging behaviour of an animal may be influenced by factors not directly associated with energy gain (e.g. predator vigilance or competing needs for other resources) as shown by Martindale [6].

In the marine environment, the foraging strategies of air-breathing diving animals are influenced by several challenges that are largely absent for terrestrial predators. While birds and bats forage in a three-dimensional environment that can constrain foraging and increase searching time in a similar manner to some air-breathing marine animals, there are considerable differences that make these two environments, and therefore the behavioural ecology of marine animals, fundamentally different (e.g. [7]). Specifically, marine predators must account for the energetic costs of having to return to the surface to breathe air between foraging dives, unlike terrestrial or aerial predators. This translates into increased energy expenditure during foraging for air-breathing marine predators as well as the need to recover oxygen stores while at the surface after deep dives. As foraging efficiency translates into increases in individual fitness, the behavioural ecology of diving animals should have evolved to use strategies to maximize energetic gains under their specific physiological and ecological constraints [8]. Within this context, air-breathing divers can be considered central place foragers in that the divers must repeatedly return to the sea surface to breathe [9]. Diving animals have also been shown to increase foraging times as foraging depth increased in order to offset transit times and maximize energy gain [10–12].

It is difficult to quantify foraging ecology of diving marine animals because (i) feeding behaviour is often performed in areas where direct observation is impossible, (ii) the tools used to derive and estimate feeding rates are complicated and logistically difficult to employ, and (iii) concurrent measurements of the distribution of prey are not easy to obtain over relevant temporal and spatial scales to foraging predators. A recent example by Benoit-Bird *et al*. [13] shows that prey distributional characteristics along with predictions from foraging theory are the causal driving factors for the distribution of a diverse group of air-breathing diving marine predators. While this provides important links, a gap still exists in being able to quantify underwater foraging behaviour concurrent with prey. Therefore, most studies of marine predator behavioural ecology have been forced to make assumptions about the potential effects of prey distribution and abundance in the absence of empirical data [7]. In reviewing foraging models for diving animals, Mori [8] predicted that predators foraging in a high-quality patch should make a large number of long duration dives to the patch. This prediction has not been rigorously tested in marine systems because of difficulties in simultaneously measuring prey patch quality and predator foraging behaviour. Mathematical simulation models, however, have revealed that prey density can affect dive time at a particular dive depth [10,14], and that feeding rates of diving predators can be affected by prey depth [12]. One recent example by Hazen *et al*. [15] indicates that blue whales maximized feeding rates and energetic gains when feeding on deep and dense prey patches (at the expense of exceeding their aerobic dive limits) rather than shallow and less dense prey patches.

Baleen whales (Mysticeti) are among the largest animals on the Earth, yet they feed primarily on animals that are smaller than their own body size by several orders of magnitude (e.g. small fish and zooplankton) [16]. As suspension filter feeders, they occupy an unlikely functional group that includes a number of shark, fish and bird species [17]. Mysticetes have evolved a suite of morphological and physiological adaptations that allow them to bulk-feed on aggregated prey [18,19]. Some of these baleen whale species comprise a family (Balaenopteridae) known as rorquals (includes blue (*Balaenoptera musculus)*, fin (*B. physalus*), sei (*B. borealis*), Bryde's (*B. edeni*), minke (*B. acutorostrata*) and humpback whales (*Megaptera novaeangliae*)) that have evolved an enlarged buccal cavity, expandable throat pleats, and an invertible tongue that act in concert to allow these animals to intermittently engulf volumes of water and prey that can exceed the mass of the whale itself [20]. This feeding strategy requires high drag and therefore demands an enormous energetic output from the whale [20,21], but it is a profitable strategy because of the large amounts of prey that can be processed during each gulp [22].

Humpback whales are unique among rorquals with respect to their morphology and variety of individual and cooperative foraging strategies [23–25]. Although humpback whales are known to feed on both schooling fish and krill, humpback whales in the Antarctic spend summer and autumn months feeding almost exclusively on Antarctic krill (*Euphausia superba*). During this period, whale distribution is primarily related to that of their prey [26,27], and krill are patchily distributed from near shore areas out to the continental shelf where spawning occurs [28]. Most humpback whales spend summers on high-latitude feeding grounds before migrating to tropical or sub-tropical breeding and calving grounds in winter. These whales generally fast during migration and breeding/calving, so they must meet their energetic demands for the entire year during the contracted feeding season. The only available information on humpback whale foraging from the Antarctic was collected towards the end of the feeding season and found that whales exhibit diel changes in diving behaviour characterized by night-time feeding dives that are significantly shallower than those during the day [29,30]. However, what is largely lacking is an understanding of how the distribution and behaviour of krill affects this observed behaviour. This information would greatly increase our understanding of how changes in the availability of prey affect the decisions of air-breathing predators to maximize rates of energy intake within the context of optimal foraging theory for central place foragers.

Using a combination of animal-borne multi-sensor recording tags and ship-mounted echosounders, we incorporate measurements of prey density and distribution with direct quantification of foraging behaviour to empirically test foraging models for air-breathing marine predators [8,10,12] that will provide insights into the ecological interactions between predators and prey. Specifically, we tested the hypotheses that: (i) whales should feed at times and in areas (depths) of relatively high prey density [22] and (ii) if prey density is consistent, feeding rates will increase with decreasing dive depths to optimize the rate of energy gain [12]. As well, we are interested in whether a relationship exists between increasing dive depth and increasing prey density (e.g. [10]). Understanding these relationships has wide-ranging implications for understanding the mechanisms that govern predator–prey interactions, the behavioural ecology and our ability to test theories of optimal foraging in aquatic air-breathing animals.

## 2. Material and methods

### 2.1. Whale tagging

**Figure 1.**
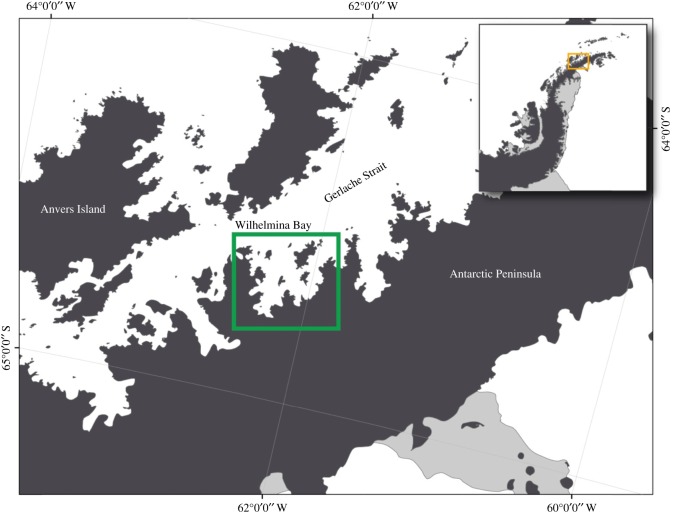
Study area of Wilhelmina Bay (open box) adjacent to the Gerlache Strait, Western Antarctic Peninsula. Black areas are land, white is water, and grey are ice shelves.

We deployed multi-sensor digital archival suction-cup tags [31] on humpback whales in Wilhelmina Bay, in the near shore waters of the Western Antarctic Peninsula (WAP) in May 2009 and 2010 (figure 1). Tag sensors included depth, temperature, 3-axis accelerometers and magnetometers that sampled at 50 Hz, and acoustics sampled at 64–96 kHz. Tags were programmed to remain on the whales for 24 h and then detach by venting the suction cups with air via a customized release mechanism. Tags were deployed from a Zodiac Mark V rigid-hulled inflatable boat with a 40 hp 4-stroke engine using a 6 m hand-held carbon fibre pole with a customized tag housing at one end. Whales were approached from oblique angles at idle or slow speeds to minimize behavioural disturbance [32] and maximize personal safety.

During daylight hours, we conducted continuous focal follows [33] of each tagged whale collecting information on group composition and behaviour as well as location. Using laser range finders and GPS, we determined the position of the tagged whale on each surface interval between dives. These positions were used to georeference the tagged whale in space and time in Trackplot, a visualization program used to characterize feeding and non-feeding dives and the depth, time and position of every feeding lunge [34,35]. At night, whale locations were determined using directional signals from a VHF antenna that was embedded in the tag. We used established methods to determine the timing and depth of feeding lunges from a combination of tag accelerometer data and acoustic flow noise on tag recordings [30,34,36]. Lunge feeding in baleen whales occurs through a series of kinematic events: rapid acceleration from high-amplitude fluke strokes followed by a significant and rapid deceleration when the whale opens its mouth and engulfs a massive volume of prey-laden water. There are often off-axis rolls or large changes in pitch associated with these movements.

### 2.2. Prey mapping and echosounder data processing

During all tag deployments, we conducted continuous and concurrent prey mapping surveys. During daylight hours, surveys were done from a Mark V Zodiac and from the *ARSV Laurence M Gould* (2009) and *RVIB Nathaniel B Palmer* (2010) using SIMRAD EK60 38 and 120 kHz split-beam quantitative echosounders. At night, surveys were only conducted from the larger ship. Echosounders were calibrated using a 38.1 mm tungsten carbide sphere following the procedure described by Foote *et al*. [37,38]. We designed prey surveys to map the distribution and abundance of krill in the vicinity of tagged whales similar to Hazen *et al*. [39] and Nowacek *et al*. [29] so that we could link the feeding lunges of whales to direct measures of prey. A clover-leaf survey pattern with the whale at the centre was performed whenever possible. In this method, the echosounders would pass by the known location of a whale as close as possible and as soon after it had dived. Prey surveys generally expanded radially out from the whale's location to a distance of 500 m. If the whale was travelling, we would perform a zig-zag survey behind the whale in its path. If the whale was not visible but could be localized with radio-tracking equipment, surveys were designed adaptively to try and survey the most likely places the whale was (or had been) feeding.

We processed echosounder data using Myriax Echoview software (v. 5.2). Krill patches were detected using the school detection module using a minimum candidate length of 10 m and minimum candidate height of 5 m [40]. The 38 and 120 kHz echograms were also visually scrutinized to ensure all krill patches were detected, including small patches. Patches were identified from the 120 kHz data using a −75 dB threshold, and the difference in scattering between the 120 and 38 kHz echosounders was used to ensure patches were composed of krill [41]. The density (both volume backscattering coefficient, *s*_v_, and mean volume backscattering strength, *S*_v_) and depth of each krill patch were then recorded and summarized by hour to compare with the distribution and location of whale feeding [39,42]. A MOCNESS (Multiple Opening and Closing Net and Environmental Sampling System) was used to confirm that acoustically detected patches consisted of krill [43] and to obtain length–frequency data of the krill in the area. Times for local sunrise and sunset were obtained from the US Naval Observatory (http://aa.usno.navy.mil/data/) for 12 May to 7 June. Transition hours that included the sunrise and sunset times were considered as daytime.

As krill concentrate into more dense aggregations and the size of the patch decreases, typical of the behaviour of schooling and swarming krill species during daytime hours [44,45], the per cent of the water column containing krill patches also decreases. We calculated the per cent area covered by krill patches by binning volume backscatter from the echosounders (*S*_v_) into 2 min (120 s) (horizontal) by 10 m (vertical) bins and comparing the proportion of bins containing krill to the proportion of empty bins for each hour of the day.

### 2.3. Data analysis

We calculated the mean depth of the krill patch/layer by hour and determined the depths of the top and bottom of the patch/layer to be able to compare this to the feeding behaviour of whales. Using only the krill data, we modelled the relationship between hourly mean krill patch depth and hourly mean krill patch density (*s*_v_) using a standard least-squares linear regression. As well, we used vertically stratified measures of krill length–frequency from MOCNESS net tows [29,46] to determine whether a relationship exists between krill size/age and depth.

Using published methods for determining feeding events from multi-sensor tag data (e.g. [30,34]), we determined the time and depth for all feeding lunges. From this, we calculated the hourly mean feeding depths and feeding rates (number of feeding lunges per hour). We then compared the hourly mean depth of whale feeding to the hourly mean depth of krill patches to look for correlation between whale feeding depth and krill patch depth. We also regressed average hourly feeding depth for all whales against average hourly feeding rates to examine foraging efficiency.

Using geo-referenced data on the locations (latitude, longitude and depth) of whale feeding lunges and prey data described above, we determined the vertically stratified prey density at the depth of individual whale feeding lunges. In ArcGIS (ESRI, Redlands, CA, USA) and R (R Core Team 2012), we buffered each feeding lunge spatially at 500 m horizontally and 10 m vertically, and temporally to 15 min before and after. Thus, we created a spatial and temporal envelope around each feeding lunge to draw a concurrent measurement of prey for our analyses and to account for any uncertainty associated with the whale's position. This spatio-temporal envelope encompasses the distance that whales travel between feeding dives as well as the time of the longest feeding dives recorded in our data [30]. Krill patches occurring within these spatio-temporal boundaries were sampled for their density (*S*_v_) and a linear regression was performed to test for a relationship between the depth of each feeding lunge and the density of krill to whether whales feed on denser prey patches with increasing feeding depths. Owing to changes in the dynamics of the prey field explained by Nowacek *et al*. [29] and Espinasse *et al*. [46] (e.g. overall krill biomass, patch location within the bay), we conducted our individual-level analyses independently for 2009 and 2010.

## 3. Results

### 3.1. Temporal krill patch dynamics

**Figure 2.**
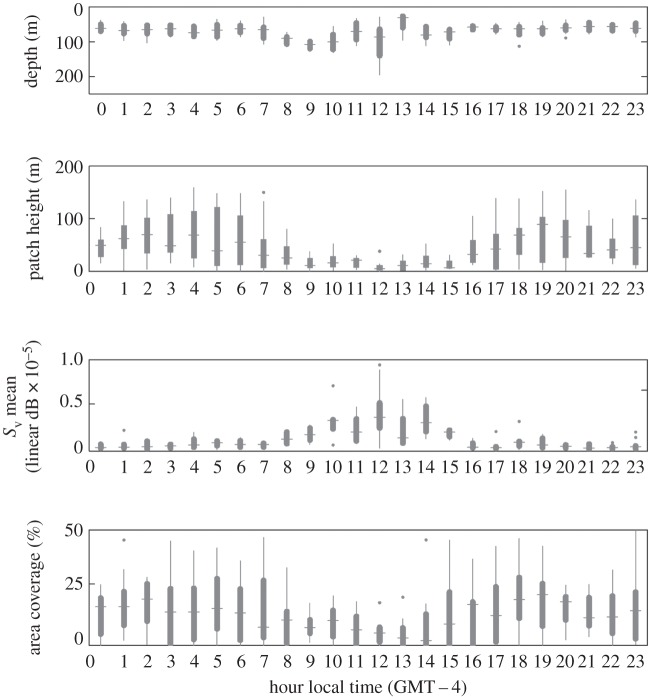
Hourly distributions of krill patch depth (m) patch height (m), density (*S*_v_) and vertical per cent area of the water column containing krill in Wilhelmina Bay in 2010 (*n* = 356 patches). Plots present the median (horizontal line), 95% lower and upper confidence bounds (thick capsules) and standard error (lines).

**Figure 3.**
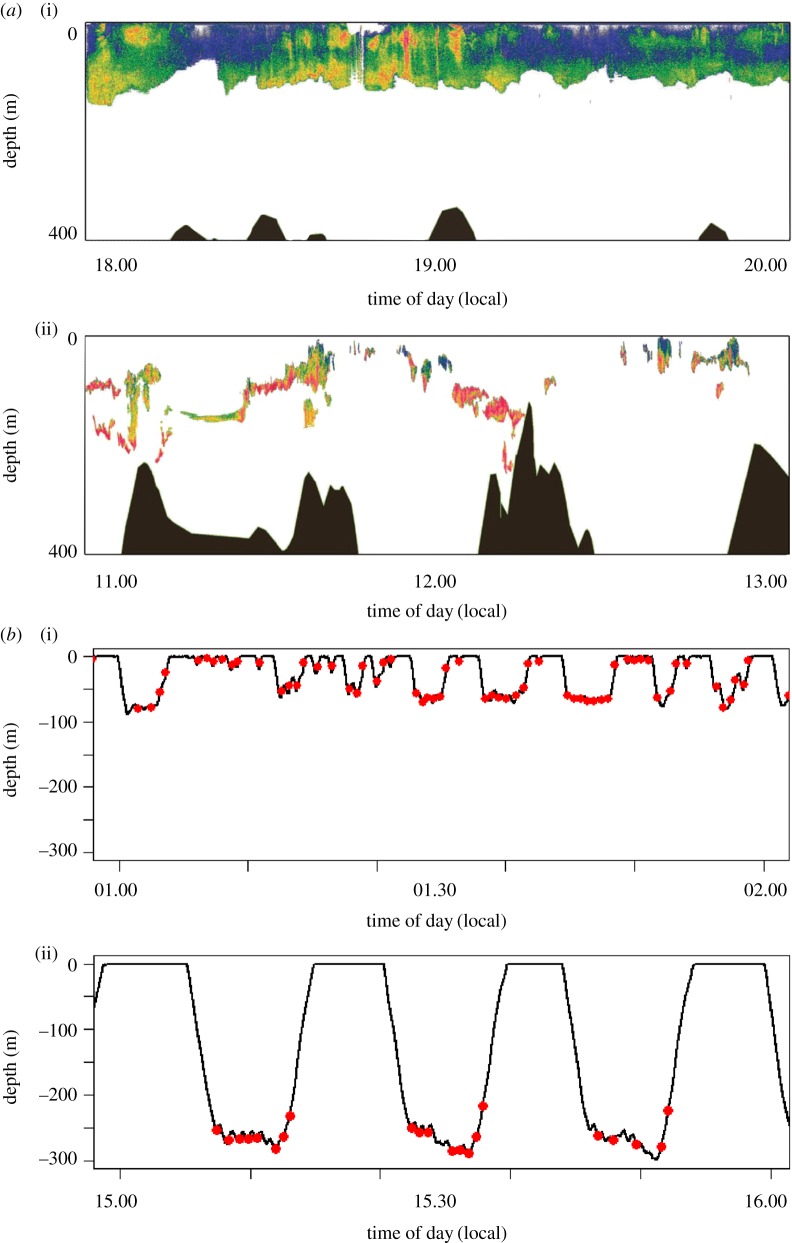
(*a*) Representative echograms showing krill patch distribution and relative density for a 2 h period at night (i) and day (ii). Data are from 13 May 2010 in Wilhelmina Bay. The colour scale from red–yellow–green–blue reflects high to low relative krill densities. Daytime patches were significantly deeper and denser, while covering a smaller portion of the water column than night-time patches. (*b*) Humpback whale dive data and feeding events for 2 h periods during night (i) and day (ii). The black line indicates whale depth and red circles indicate lunge feeding events. During the night, whales make more repeated shallow dives with fewer feeding lunges per dive while during the day whales make fewer, deeper dives with more feeding lunges per dive.

We collected 274 h of echosounder data concurrent with tag deployments. Krill patches, or the krill layer, occurred at significantly greater depths during day than at night (two-sample *t*-test, d.f. = 62, *p* = 0.001). Based on data from 183 krill patches, we found that the mean daytime patch/layer depth was 86 m (95% confidence interval = 77–96 m), while the mean night-time depth was 64 m (95% confidence interval = 62–67 m). Patches were significantly shorter in vertical height (two-sample *t*-test, d.f. = 62, *p* = 0.001) during the day than at night. Mean daytime patch height was 18 m (95% confidence interval 13–22 m), and mean night-time patch height was 59 m (95% confidence interval 51–67 m). Patches were also significantly denser during the daytime than night-time (two-sample *t*-test, d.f. = 62, *p* = 0.001). Mean daytime *S*_v_ was −55.9 dB (95% CI −55.87 to −54.11), and mean night-time patch *S*_v_ was −60.54 dB (95% CI −61.70 to −59.62). Similarly, the percentage of the water column covered with a detectable krill patch was significantly lower during daytime hours than during night-time hours. In summary, krill in daytime were deeper, denser and more compact than at night (figures 2 and 3*a*).

**Figure 4.**
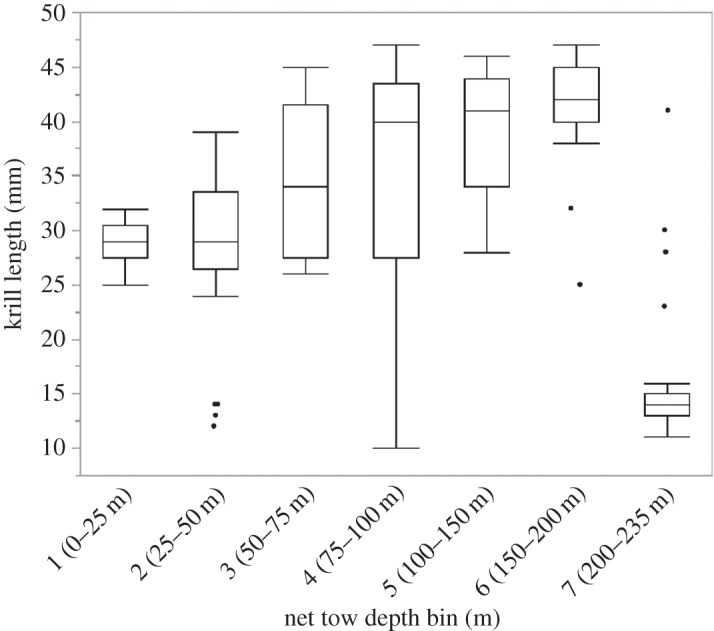
MOCNESS net tow length–frequency distributions for Antarctic krill (mm). Box plots show the mean, standard deviation and individual outliers of krill length frequencies for each depth bin (m).

**Figure 5.**
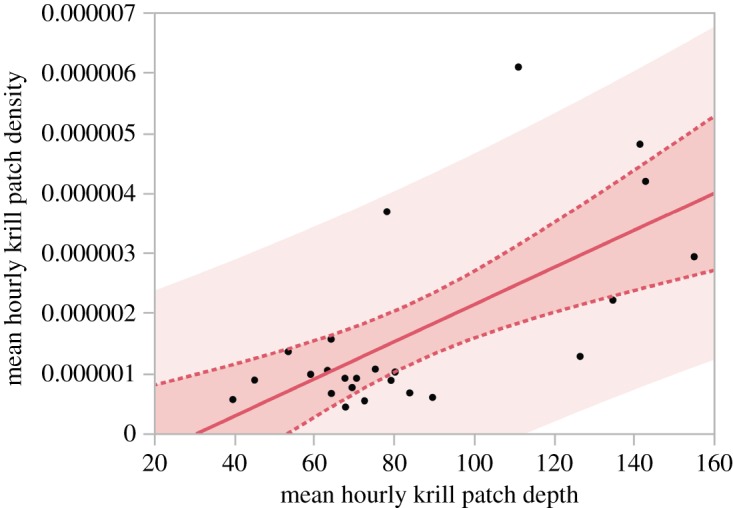
A linear regression of hourly mean krill patch depth (m) versus mean krill patch density (*S*_v_). The model shows a significant positive relationship (*p* = 0.005, adjusted *R*^2^ = 0.40), where mean hourly krill patch density increased as mean hourly krill patch depth increased.

From MOCNESS tows, we found a single unimodal size/age class (4.2 ± 0.6 cm) of *Euphausia superba* that dominated the sample [29] (table 1). When examined vertically, we found an increase in the average size of krill with increasing depth to 200 m (figure 4). Below this depth, and below the feeding depth of the whales, a single larval krill cohort was found in very low abundance. Using a Tukey–Kramer HSD comparison of means, we found a significant increase (at *p* < 0.05) in mean krill length with increasing patch depth. The linear regression of mean hourly krill patch depth and mean hourly krill patch density revealed a significant positive relationship (*p* = 0.005, adjusted *R*^2^ = 0.40), where mean hourly krill patch density increased as mean hourly krill patch depth increased (figure 5).

**Table 1.** Tukey–Kramer HSD comparison of mean krill lengths from depth-stratified MOCNESS tows. Similar letters reflect statistically similar mean krill lengths between depth bins. Group A contains similar-sized krill from 100–150 and 150–200 m. Group B from 50–75, 75–100 and 100–150 m. Group C from 0–25, 50–75 and 75–100 m. And group D from 0–25, 25–50 and 75–100 m.

net depth (m)	mean krill length (mm)				
150–200	41.9	A			
100–150	38.9	A	B		
50–75	34.7		B	C	
75–100	34.2		B	C	D
0–25	28.8			C	D
25–50	28.7				D

### 3.2. Whale feeding and krill patch metrics

We tagged nine adult humpback whales in Wilhelmina Bay between 2 April and 2 June (2009 *n* = 4, 2010 *n* = 5) and collected a total of 202.24 h of tag data. Deployments ranged between 18 h and 45 min and 25 h and 38 min. A total of 2252 feeding dives were recorded between the hours of 14.00 and 08.00 local. Very few feeding dives occurred between 08.00 and 14.00, local time, rather these were exploratory dives as discussed by Friedlaender *et al*. [30]. An example of the diving behaviour of feeding humpback whales during different times of the day in relation to the availability of krill is shown in figure 3*b*. Whales fed deeper and executed more lunges per dive in afternoon (14.00–16.00) than during night-time hours when they generally performed shallow feeding dives with one to two lunges per dive [30,34].

**Figure 6.**
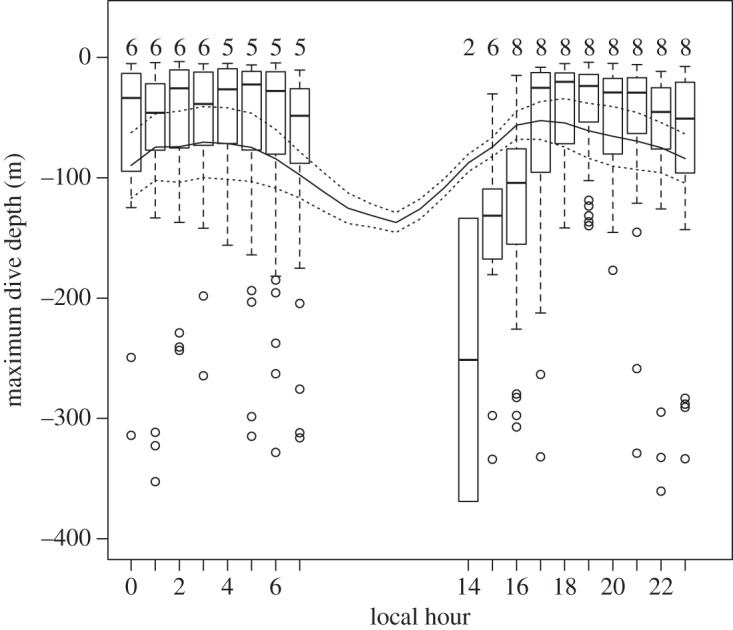
The correlation (*R*^2^ = 0.41) between humpback whale hourly feeding depth and mean hourly krill patch depth. Box plots indicate the mean, standard error and outliers for whale feeding depths (m) during each hour of the day. The number above each plot indicates the number of tagged whales that were feeding during that period. The continuous line represents the moving average mean depth of the krill (m), and the shaded lines represent the top and bottom of the depth of the krill patch or layer. Hours with no box plots or numbers indicate hours where no feeding occurred (tags were deployed on eight whales during the hours with no feeding).

Tagged humpback whales lunged from 1 to 13 times per feeding dive. We found a positive relationship between the number of lunges on a given dive and the maximum depth of the dive (adjusted *R*^2^ = 0.61). We found that when whales began feeding in late afternoon hours, these depths were deeper and were concurrent with when krill were deeper in the water column and migrating vertically (figure 6). Throughout the night, from 17.00 to 07.00, both the whale feeding depth and mean krill patch depth remained relatively stable. Whale feeding continued through the 07.00 h as krill began to migrate deeper, concurrent to increased patch density and decreased patch height. By 08.00 all whale feeding ceased while krill depth continued to increase.

**Figure 7.**
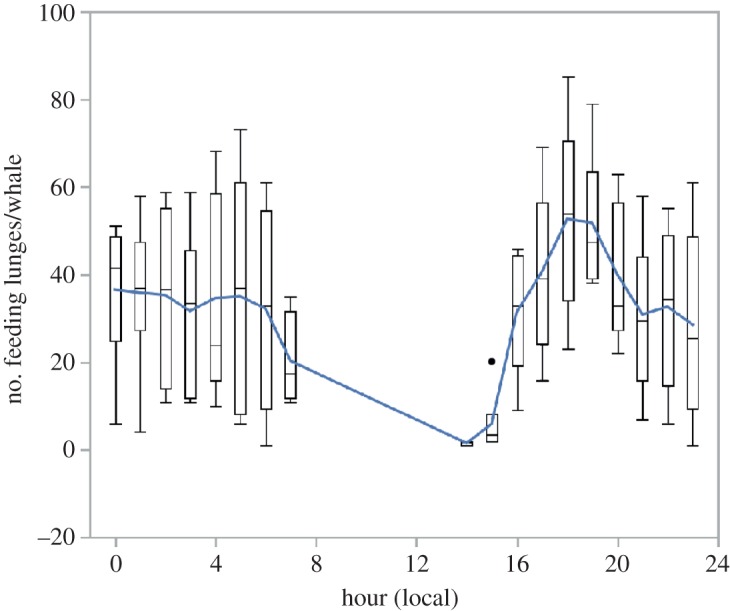
Hourly feeding rates of tagged humpback whales represented by box plots and trend line. The box plots indicate the mean, standard error and outliers for the number of feeding lunges performed by tagged whales during each hour of the day. The line represents a moving window average of feeding frequency.

**Figure 8.**
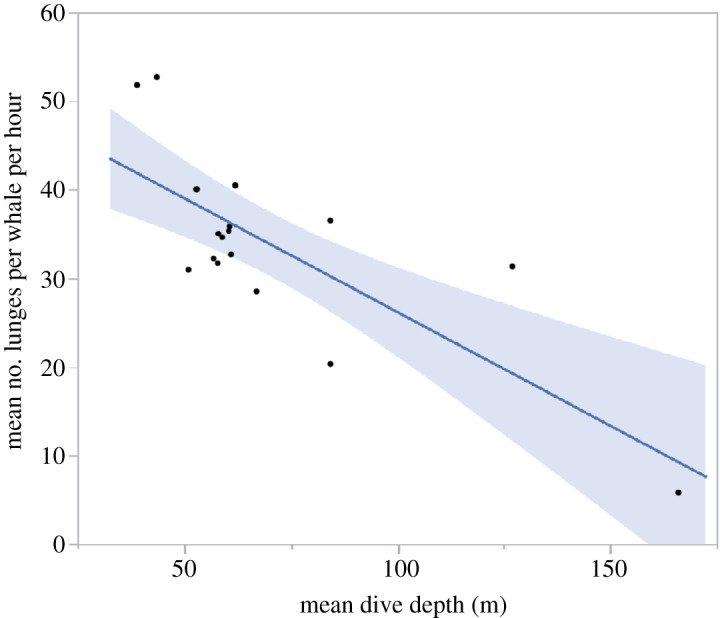
Least-squares regression relating the mean feeding rates (number of lunges per hour per whale) to the mean depth of feeding lunges during each hour. The relationship shows a significant (adjusted *R*^2^ = 0.59, *p* < 0.003) decrease in feeding rates with increasing feeding depth.

Feeding rates, the numbers of lunges per hour, for all animals are shown in figure 7. Similar to dive depth, we find that when whales began feeding in late afternoon, foraging rates were lowest and increased into the evening and then remained relatively stable throughout the night until whales stopped foraging in the morning. We found a significant (*R*^2^ = 0.59, *p* < 0.003) relationship between feeding rates (mean number of feeding dives per whale per hour) and feeding dive depth with the highest feeding rates occurring at the shallowest feeding depths (figure 8).

**Figure 9.**
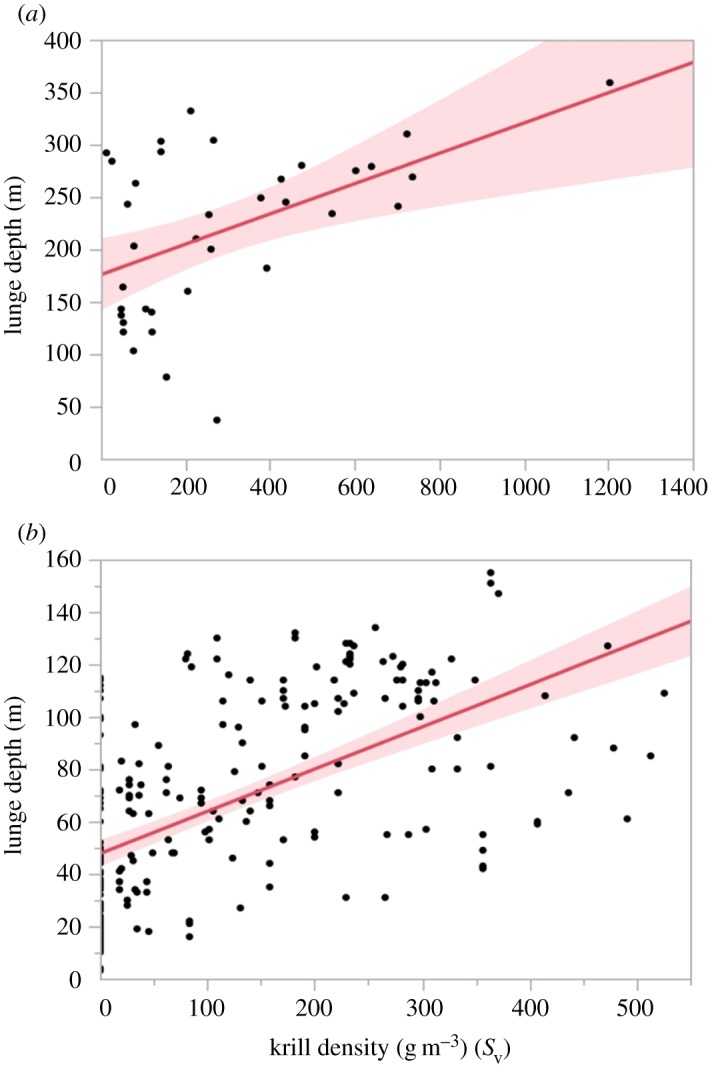
Least-squares regression relating the depth of whale feeding lunges with concurrent krill densities in g m^–3^ (*S*_v_) in 2009 (*a*) and 2010 (*b*). Both years indicate a positive relationship between increased depth of individual whale feeding lunges and increased krill density. In 2009, adjusted *R*^2^ = 0.25; in 2010, adjusted *R*^2^ = 0.33.

We collected concurrent measurements of krill patch density spatio-temporally linked with 36 feeding lunges in 2009 (*n* = 2 whales) and 243 feeding lunges in 2010 (*n* = 2 whales). In both 2009 (*R*^2^ = 0.25) and 2010 (*R*^2^ = 0.33), we found a positive relationship between whale feeding depth and increasing krill density (figure 9). In 2009, our measurements included foraging bouts that were deeper than those measured in 2010 and also contained krill densities that were greater in 2009 than in 2010. Thus, for individual lunges performed within or across dives, the depth of whale feeding increased with increasing krill density.

## 4. Discussion

All air-breathing diving animals face the conflicting demands of minimizing oxygen use during diving and the energetically costly manoeuvres associated with capturing prey at depth [48]. Thus, in order to optimize the energetic efficiency of foraging, animals should increase feeding rates where patch quality and density are high. However, when prey is not easily accessible, density is not the only important factor and the costs associated with increased travel time to patch depth (e.g. oxygen use, recovery time) must be considered. Intermittent bulk-filter feeders, such as lunge feeding baleen whales, face an additional challenge, as each prey capture event is energetically costly due to the high drag associated with lunging [49]. Therefore, lunge feeding baleen whales are critically dependent on prey density to maximize the number of prey captured in a single feeding event. However, dense prey often migrate to depths [29] that are at the limits of measured whale feeding [30] to avoid predation during the day, but then migrate upwards towards the sea surface and disperse at night to feed [50]. As a result, these whales may choose between two distinct foraging strategies: (i) dive deep at greater cost to feed on higher density prey or (ii) wait for more easily accessible, lower quality prey patches.

With respect to the hypotheses we set out to test, we found an interesting combination of results. Surprisingly, the whales in our study did not feed during times and in depths of the highest prey density as predicted by Goldbogen *et al*. [22]. If this prediction were true, whales would have fed during the day when krill were densest and found deep in the water column (though still accessible to diving humpbacks *sensu* [30]). Instead, whales prioritized rest during the daylight hours. During feeding, as dive depth increased so too did the number of feeding events per dive as demonstrated by Ware *et al*. [34], but more importantly feeding rates decreased with increased dive depth as predicted by optimal foraging theory and Doniol-Valcroze *et al*. [12]. While the number of lunges per dive decreased on individual shallow dives, the overall feeding rate (lunges/hour) was the greatest during these times. During feeding bouts however, dive depth varied and we found that within the range of depths that whales fed during the night, the deeper they fed, the denser the prey was that the whales targeted (as predicted by Mori [10]). Given the overall pattern in feeding rates, we believe that the latter strategy of foraging on shallower prey must help to maximize (or at least maintain successful) foraging rates as the distance between prey and the central place (sea surface) increased. We posit that these multiple-step foraging decisions in Antarctic humpback whales should increase overall feeding efficiency by minimizing transit costs and maximizing prey intake. These data are particularly useful in understanding how the distribution and density of prey affect the behavioural ecology of air-breathing, marine central place foragers.

In the waters around the WAP, humpback whales exploit the diel vertical migration of krill in the water column [46] that results in decreases in patch density but increases the overall area of the water column occupied by krill. During the day, the krill are found in more discrete, denser patches; whereas at night, these same krill disperse into a more consistent layer. This suggests that diving deeper to locate fewer, denser, but more sparsely distributed krill patches may not be as efficient as waiting for krill to move closer to the surface and disperse. Indeed, our results show that whales forage when krill are closer to the surface, where they expand and both the horizontal area and vertical height of krill patches are increased. This type of krill distribution presumably increases whale foraging efficiency by decreasing search time and increasing the probability of detecting and locating prey. Moreover, whales should benefit from a larger search volume that is closer to the sea surface, and should be able to optimize the management of oxygen stores more efficiently by decreasing lunge frequency and dive duration [12]. In this way, whales can couple a large proportion of the filtration cycle (time in between lunges) with the respiratory cycle (momentary breaths at the sea surface in between dives) to forage continuously [34]. Such a strategy contrasts from deep foraging dives, which require increased surface recovery time to replenish oxygen stores [51]. In this case, oxygen is acquired at the surface with diminishing returns, and therefore recovery times increase rapidly with the lengthening of the preceding dive. If this were a linear relationship (twice the dive duration = twice the surface recovery), then there would be little incentive for divers to minimize dive duration because all strategies would result in the same total recovery time over the entire feeding bout.

The krill on which the whales in our study were feeding were part of an extremely large and dense aggregation [29]. While the majority of the krill were adult sized [46], we did find an increase in the average size of individual krill with increasing depth in the feeding range of the whales (figure 4). As well, there was no indication that the composition of the krill aggregation changed during diel vertical migration [29,46]. Thus, it appears that the whales in our study were also foraging on larger sized krill as they increased their feeding depths, which may be another means by which feeding deeper could still be as efficient as shallow feeding. Future work could investigate whether the energetic content of prey varies both with individual size and depth to determine if this may be yet another means by which whales maximize their energetic gains.

Our results indicate that humpback whales in Antarctica in autumn generally begin feeding once krill have migrated to the upper portions of the water column. As individual whales vary their feeding depths and forage deeper (typically in crepuscular periods) [30], they do so on significantly higher densities of krill. This density-dependent relationship that dictates the behavioural ecology of these whales appears to be conserved regardless of time of day: whales target deeper and denser krill within the depth ranges where krill migrate to at night. Furthermore, our results suggest that humpback whales foraging late in the feeding season around the WAP do so in a manner that is consistent with optimal foraging to maximize energy intake in two ways: (i) by generally feeding at shallow depths during night-time hours to increase feeding rates when krill are shallow to increase feeding rates and (ii) when they do have to dive deeper, targeting denser prey patches that contain larger individual krill. This multiple-step foraging decision in a central place forager demonstrates ways in which the behavioural ecology of these predators acts to optimize overall efficiency by minimizing diving costs and maximizing prey intake. These data reveal a previously unrecognized level of complexity in predator–prey interactions in marine predators and underscores the need to study foraging behaviour and prey distribution simultaneously.

As demonstrated by Richman & Lovvorn [52] for other diving marine animals, partitioning prey resources by size and depth can reduce competition between different sized predators. However, shifts in the size–depth structure of these prey resources can greatly alter the relative ability of each predator to persist. Using the methods described here, direct measures of the foraging behaviour and decisions of humpback whales can now be combined with similar studies of other predators of varying size (e.g. Antarctic minke whales [53], crabeater seals [54] and Adelie penguins [55]) to test whether sympatric species partition resources to reduce the potential for competitive interactions in this (and other) marine ecosystems.

Our study also presents a relatively novel approach to collecting quantitative prey data in spatio-temporal concordance with fine-scale measurements of air-breathing animal feeding behaviour. This combination of using multi-sensor animal-borne data logging instruments with traditional means to describe the feeding environment can be a powerful tool for future studies aimed at understanding how the behavioural ecology and ecological interactions of a wide number of marine predators are affected by their environment. This has been a logistical challenge for researchers focused on studying diving aquatic animals where the opportunity to continuously observe animals and their environment (as can be done in terrestrial systems) is limited.

While our results provide valuable and useful information, there are limitations that could potentially affect our findings. Studies focused on free-ranging, wild cetaceans are often limited in sample size due to the logistical constraints associated with this type of work. Combined with the remote location and time of year of our study, we have a limited sample size from which to draw our conclusions. While we present data from two field seasons, it is possible that there is greater temporal variation in the behaviour of both whales and krill than what we have measured. Likewise, humpback whales are known to exhibit remarkable individual variation in their feeding behaviour (e.g. [24,25]), and our data may represent individual foraging strategies rather than those of the population as a whole. The methods we use for measuring the distribution and abundance of krill are also limited in that our echosounders have a very narrow beam-width, essentially like shining a flash-light in a dark room. Therefore, while we collected a significant amount of data, we are not able to comprehensively assess the complete environment around feeding whales in the time and places where feeding occurs. Since we cannot simply map an individual patch of krill over the period of time when it is being fed upon by a whale, it is also difficult for us to determine the capture efficiency and the precise consumption rates of the whales. While this information would greatly inform optimal foraging models focused on diving marine predators, it is beyond the scope of this paper.

Here, we used concurrent measurements of both prey and whale behaviour to demonstrate that humpback whale feeding around the WAP was affected significantly by both the depth and density of krill patches in the water column. Surprisingly, these whales did not feed during daylight hours when krill were found deeper and in denser patches, as is commonly observed in other krill feeding baleen whales in the eastern North Pacific [56–59]. Nevertheless, the observed behaviour of whales tending to forage when prey patches are closer to the surface is also seen in some toothed whale species that appear to optimize encounter rates with deep-scattering layer prey (squid and fish) by limiting foraging dives to night-time hours [60]. In this study, we show that, in addition to prey patch quality (density), prey accessibility can play an important role in driving foraging behaviour. Therefore, our results underscore the importance of both prey patch quality and accessibility (including the probability of detecting prey) for optimal foraging [8], yet most optimality models focus on these elements independently (e.g. [14]). Future research should focus on the interaction between prey depth and density and how it relates to foraging performance over greater temporal scales.

Understanding the behaviours that optimize the foraging strategies of baleen whales, the largest extant filter feeding animals, provides an example for broader comparative studies of the evolutionary forces that shape the behavioural ecology of animals across a range of taxonomic groups, scales of body size and environments. For example, our findings on how prey density and depth affect the foraging behaviours of whales can be compared with studies of evolutionarily related terrestrial herbivores (e.g. [61]) to gain insights into the differences that have driven optimal foraging strategies in both terrestrial and marine systems. Bulk-filter feeding as a foraging strategy has evolved independently and repeatedly among a diverse group of animals including the largest vertebrates (e.g. basking and whale sharks, and manta rays), fossil or living, leading to a 100 Myr dynasty of giant filter feeding bony fishes [62]. Being able to quantify the feeding performance of baleen whales can offer insights for comparisons among these groups that can lead to a greater understanding of the behavioural ecology of this successful foraging strategy.

## Data accessibility

All data are available through the mce.ml.duke.edu RAID server.
